# Analysis of biomolecules in cochineal dyed archaeological textiles by surface-enhanced Raman spectroscopy

**DOI:** 10.1038/s41598-021-86074-9

**Published:** 2021-03-22

**Authors:** F. Celis, C. Segura, J. S. Gómez-Jeria, M. Campos-Vallette, S. Sanchez-Cortes

**Affiliations:** 1 Laboratorio de Procesos Fotónicos y Electroquímicos, Departamento Disciplinario de Química, Universidad de Playa Ancha, Casilla 34-V, Valparaíso, Chile; 2 Faculty of Sciences, Department of Chemistry, University of Chile, P.O. Box 653, Santiago, Chile; 3 Instituto de Estructura de La Materia, IEM-CSIC, 28006 Madrid, Spain

**Keywords:** Other nanotechnology, Biophysical chemistry, Bioanalytical chemistry

## Abstract

SERS spectroscopy is successfully employed in this work to reveal different components integrating the cochineal colorant employed for dying archaeological textile samples from the Arica Region in North Chile. This analysis was done by *in-situ* experiments that does not imply the material (colorant and biomolecules) extraction. The spectroscopic analysis of the archaeological textiles by SERS reveals the presence of bands attributed to carminic acid and nucleobases, mainly adenine and guanine. The identification of these biomolecules was also verified in raw cochineal extract and in cochineal dyed replica wool fibers fabricated by us following ancient receipts. The effect of Al on the complexation of carminic acid and other biomolecules was also tested in order to understand the changes induced by the metal interaction on the colorant structure. This study revealed that Al can also complex biomolecules existing in the cochineal extract. In particular, guanine residue seems to interact strongly with the metal, since SERS bands of this residue are enhanced. Furthermore, a theoretical analysis on the interaction of carminic acid and a silver surface was also performed in order to better understand the interaction mechanism between carminic acid and a metal surface that leads to the final SERS spectrum. The results of the present work will be very useful in the identification of different molecules and metal complexes that may be forming part of the cochineal colorant found in archaeological materials.

## Introduction

**Figure 1:**
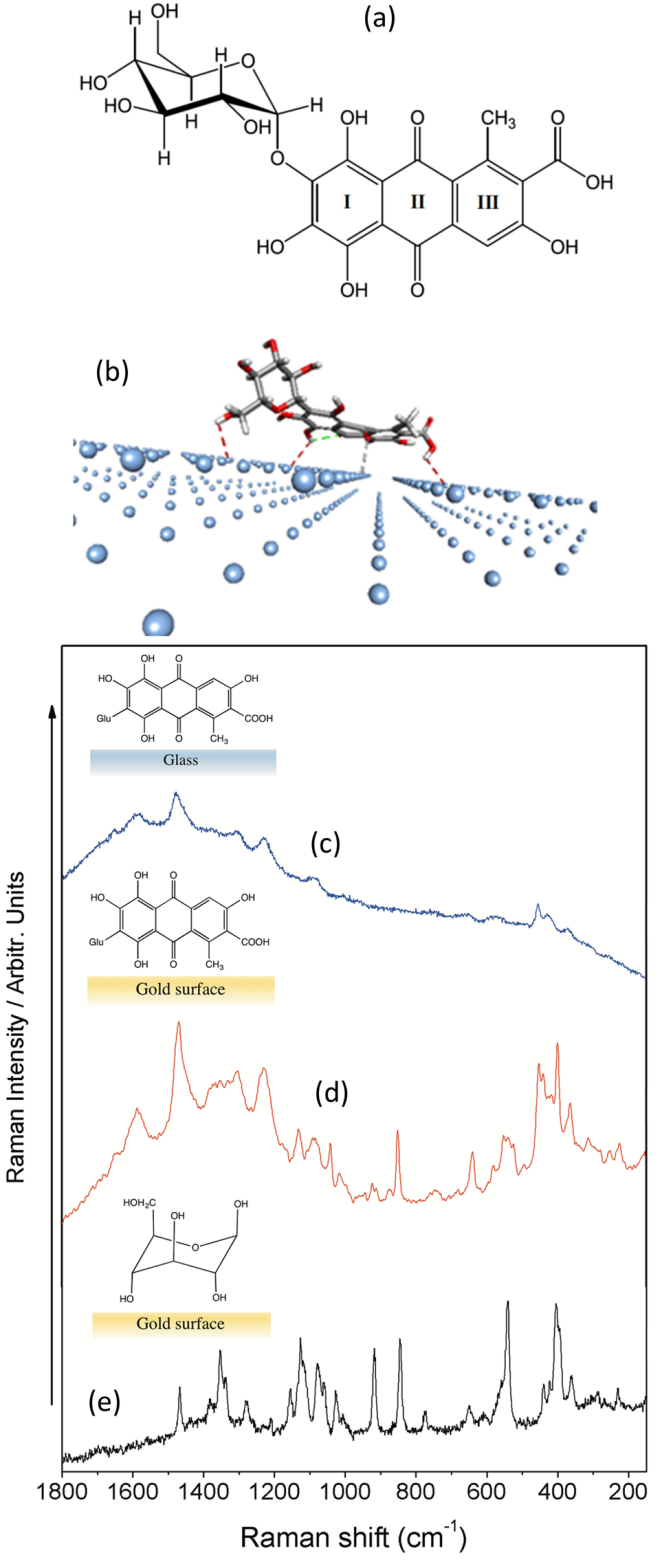
Structure of carminic acid (**a**) and scheme of the interaction of carminic acid with an Ag surface (**b**). Raman spectra of carminic acid on glass (**c**) and on a Au surface (**d**). Raman spectrum of glucose on Au surface (**e**).

Cochineal is one of the most important red colorants used for dying textiles. Specifically, the Mexican Cochineal refers to the crude material obtained from the dried bodies of the plant parasitic hemipterous insect of the same name (*Dactylopidae cocus)*, which can be found in the tuna cactus (*Opuntia spp.*). Carminic acid (Fig. 1a**)** is the most abundant colorant component of Cochineal colorant, and represents the 15–20% of the total insect weight^1^. Cochineal trade gave rise to one of the main historical markets between America and Europe until the synthesis of new red dyes in the XIXth century^1^. In fact, it was employed to dye textiles in the antiquity. The potential presence of many other biological constituents of the insect body in the carmine colorant difficult its detection in archaeological materials. In general, cochineal fabrication implies a process of purification by alkaline treatment which renders a great variability in the composition. This extract is generally named as cochineal extract. Moreover, this variability is further increase because of the different origin of the harvested insects^2^. An important step in the use of cochineal dyes is the use of metals, usually Al, leading to the formation of Al lakes that also favor the immobilization of carminic acid on textile fibers. In general *Carmine* refers to a metal coordination complex or lake involving aluminum and carminic acid. This colorant is produced directly from the cochineal extract without a further purification^2^. Therefore, carmine is usually a very complex material integrated by a large list of different colorants and other biological molecules as well as fragments of the insect body, waxy exudates and residue from the environment, although carminic acid is the main component of this mixture. In fact, chromatographic methods have revealed the presence of other minor colorants in the cochineal extract, such as kermesic and flavokermesic acids, as well as other unidentified compounds^3^. All these molecules can also form a complex with the metal used in the fabrication of the carmine lake. The presence of cochineal and/or carmine in textile archaeological materials is very frequent in samples found in the American continent^4, 5^. In fact, the detection of this colorant in many ancient American textiles was reported in recent works^6–9^.

Analytical methods based on chromatography^10–12^ are very specific, although they require the extraction of colorant from the material and a subsequent pretreatment previous to the final analysis. Spectroscopic techniques can be applied in the *in-situ* analysis of cochineal. For instance, the *in-situ* analysis of carminic acid can be performed by FORS (Fiber Optics Reflectance Spectroscopy). However, the analysis derived from this technique is not univocal because it provides broad bands that are difficult to assign^6, 13^.

Raman spectroscopy has been largely employed in the analysis of colorants in art and archaeological materials^14, 15^. However, the intrinsic low intensity Raman scattering and the large fluorescence emission usually induced by biomolecules, avoids, in the majority of cases, the analysis of organic dyes. To overcome this issue, the use of metal nanoparticles, mainly made of gold and silver, increases the Raman cross section of molecules thanks to the SERS (Surface-Enhanced Raman Scattering)^16^. SERS has been successfully employed in the identification of natural colorants, including cochineal and other red related lakes, in archaeological materials in general^15, 17–20^ and in textile fibers in particular^21, 22^. Metal nanoparticles have also the advantage of inducing fluorescence quenching. Another advantage of SERS is that it allows the analysis of colorants without extraction of the pigment from the fibers textile in methods implying the deposition of metal nanoparticles on the fiber^23^ or by *in-situ* photoreduction of Ag^+^^24^.

The spectroscopic analysis of cochineal is advantageous but it poses significant challenges: (a) the rather weak interaction of carminic acid with metal interfaces, a required process for SERS intensification; (b) the residual fluorescence that can still be detected in samples containing cochineal^25^; (c) the different structures adopted by carminic acid in the mixture by the possible ionization of the carboxylate and phenol groups^25^; (d) the metal complexation that also leads to a wide spectrum of vibrational bands in cochineal extracts; and (e) the intrinsic heterogeneity of cochineal colorants that are accompanied by a large list of organic material and biomolecules from the insect body. Therefore, it is difficult the elaboration of a complete carminic acid and cochineal Raman database that could be used for the colorant identification.

The problem of the heterogeneity in cochineal dyestuff was not addressed conveniently so far. Spectroscopic and chromatographic analysis of textile extracts or cochineal extracts where not able to identify unknown bands that could be originated from biological components of the insect body^3, 26^. In addition, the metal complexation of carminic acid in lakes was not studied in depth. In this context, SERS spectroscopy can be proposed as a very sensitive technique that can afford more insight on the analysis of the complex archaeological samples where carminic acid is present.

For all the above reasons, the main goals of the present work were (a) the identification of biomolecules in cochineal dyed textile fibers and (b) the analysis of the effect of Al as mordant in the immobilization of the colorant as well as other biomolecules naturally occurring in archaeological materials onto the wool fiber. To accomplish these tasks, a spectral Raman and SERS analysis was done in historical textile samples and replica dyed wool fibers. These replica samples were prepared by employing the same procedures used in the past and using wool threads directly dyed with commercial carminic acid and the cochineal extracted by us from commercial insects. The spectral *in-situ* analysis was also completed with a spectral investigation of carminic acid and D-glucose, as well as a theoretical study on the interaction with metal surfaces in order to improve the band assignment of these materials.

For the best of our knowledge, this is the first time that Raman is employed in the analysis of biological molecules potentially existing in cochineal dyestuff and that the effect of Al complexation on cochineal is tested by Raman and SERS. Furthermore, the use of star-shaped silver nanoparticles (AgNPs), following the nanofabrication developed at our laboratory, was able to induce strong enhancement the SERS sensitivity leading to more successful results. The information reported in the present analysis will be helpful for specialists that can infer about the dying techniques used by ancient cultures, mainly in Central and Southern America where red colorants were frequently employed.

## Experimental

### Materials

Carminic acid and Al_2_(SO_4_)_3_ (analytical standard) were purchased from Sigma-Aldrich and used as received. Calf thymus DNA (ct-DNA) and poly(dG-dC).poly(dC-dG) (pGC.pCG) were purchased from Sigma Aldrich. D-glucose (Glu) powder was purchased from ThermoFisher and used as received. Dried and grinded cochineal insects employed in the dyeing wool-cochineal process were purchased from Telasybastidores (Pertoca—Valparaíso Region, Chile). The stock solutions were prepared in ultrapure water (conductivity 18,2 µS/cm). Commercial white wool was purchased from a textile shop in Madrid, and it was used for the dying process without any treatment and used as received. Silver nitrate, trisodium citrate and absolute ethanol were purchased from Merck, while hydroxylamine hydrochloride and hydroxylamine solution (50% w/w in water) were purchased from Sigma Aldrich. The thin sheet of gold was specially prepared in the facilities at the Faculty of Physical and Mathematical Science (University of Chile) in order to perform the Raman measurements of carminic acid and Glu. Archaeological samples correspond to the Formative Period of the Arica Culture and were provided by the Museo Chileno de Arte Precolombino in Santiago de Chile.

### Preparation of a wool-cochineal replicas

Textile fibers dying was accomplished by following the protocol published by Maier et al.^27^. According to this method, three dried cochineal insects (140 mg approximate of total weight) were placed into the agate mortar and grind treated until obtaining a fine powder. Then, this powder was deposited in a flask with 5 mL of ultrapure water. The solution was stirred and heated to 90 °C and after that, a piece of 1 cm of length of commercial white wool was added. The solution was stirred during 30 min without heat. After this dying procedure, the resulting red wool was washed with ultrapure water and dried for 12 h. A second piece of white wool was dyed with the same methodology, with the difference that 100 mg of Al_2_(SO_4_)_3_ was added before soaking the white wool in the cochineal solution following previously reported recipes^28, 29^. Both pieces were used in dry conditions before adding a drop of star-shaped NPs suspension for the SERS experiments. Both samples displayed a final pH of 5.2.

### Synthesis and characterization of silver nanoparticles

Silver nanostars (AgNS) were prepared by reduction of Ag^+^ with neutral hydroxylamine and sodium citrate in two steps, according to the method reported previously by us^30^. First, 500 μL of NaOH 5 × 10^–2^ M was mixed with 500 μL of hydroxylamine 6.02 × 10^–2^ M. Then, 9 mL of AgNO_3_ 1 × 10^–3^ M was added dropwise to the solution. The resulting solution was stirred during 5 min. Finally, 100 μL of 4.13 × 10^–3^ M trisodium citrate was added. This suspension was stirred for 15 min. Spherical citrate AgNPs were prepared according to the Lee and Meisel chemical reduction method^31^.

### Samples for Raman

The Raman spectra of carminic acid and Glu were obtained by depositing an aliquot of the carminic acid solution (10^–3^ M) on an Au thin surface. The gold surface was prepared by deposited Au film in Argon plasma on glass substrate by sputtering method. A laser at 785 nm laser line was employed as excitation with a final power at the sample of 1mW. Final spectra were the result of 5 accumulations registered with an integration time of 20 s and a spectral resolution set at 4 cm^−1^.

### Samples for SERS

Samples for SERS spectra were prepared by depositing 100 μL of concentrated AgNS on each fiber. The concentrated AgNS suspension was prepared by centrifugation of the AgNS prepared as described above (5 min at 13,000 rpm), thus removing the supernatant until concentration in 10% of the initial volume. Each piece of wool was deposited inside the well of a microscope slide. After deposition of the concentrated AgNS suspension, the water solvent was evaporated at room temperature without heating. Spectra were registered three hours later on different points of the sample. Under these conditions high spectral reproducibility batch to batch was obtained for each sample.

### Instrumentation

#### Raman

A micro-Raman system (RM1000, Renishaw) equipped with the 785, 633 and 514 nm laser lines, a Leica microscope and an electrically cooled charge couple device (CCD) detector was used to register the Raman and SERS spectra. The laser power on the Ag colloidal coated samples was ca 2 mW. The instrument was calibrated using the 520 cm^−1^ band of a Si wafer and a 50 × objective. Its resolution was set to 4 cm^−1^. Spectral recording conditions and the choice of the laser line used were selected in order to avoid sample degradation. In this sense, the 633 nm laser line was used in the SERS analysis of the wool and the red dyed wool, while the line at 785 nm was employed for the solid samples in order to avoid the fluorescence emission. The 514 nm line was employed to measure the SERS of nucleic acids. SERS spectra were recorded by averaging 5 scans using an integration time of 20 s and a spectral resolution at 4 cm^−1^.

### UV–visible Absorption Spectroscopy

UV–vis absorption spectra of AgNS and AgNPs were recorded from on a Shimadzu 3600 spectrometer by using quartz cells, 1 cm optical path. Samples were diluted 30% in MilliQ water (volume/volume). The UV–visible spectrum of AgNS shows two maxima at 384 and 418 nm, while a large extinction with a broad tail towards the red-near infrared is seen at higher wavelength which is due to the presence of AgNS with different morphologies.

### Transmission Electron Microscopy (TEM)

TEM images were obtained with an Inspect S50 (FEI, Hillsboro, Oregon, OH, USA) scanning electron microscope. The analysis was performed with 20 kV as High Voltage. Extinction spectra of colloids were recorded on a Shimadzu 3600 spectrometer (Shimadzu Corp., Kyoto, Japan) equipped with a PMT for light detection in the UV–visible range and an InGaAs detector for the NIR was employed to obtain the plasmon extinction spectra. Samples were placed in quartz cells of 1 cm optical path, after dilution to 30% in MilliQ water (v/v).

#### Theoretical calculations

The carminic acid geometry was optimized by using the B3LYP/6-31 g(d,p) calculation level in Gaussian 09 software^32^. The silver metal surface model, Fig. 1b, was already described^33^ and the HyperChem software (Hypercube, Inc.) was used for the calculations. To optimize the carminic acid-Ag geometry we have employed molecular mechanics at the OPLS level by maintaining fixed the Ag layer geometry.

## Results and discussion

### Raman spectrum of carminic acid

The Raman spectrum of carminic acid in solid state was obtained by depositing a solution of this compound on glass (Fig. 1c**)** and on a rough gold surface (Fig. 1d**)** in order to reduce the intrinsic fluorescence emission and improve the Raman signal. The analysis of spectra reveals the appearance of bands with an intensity variation depending on the sample preparation (glass or Au surface). Raman of carminic acid in solid state presents the characteristic Raman bands of the protonated molecular form. In general, the most intense bands of the spectrum correspond to the aromatic anthraquinone moiety, undergoing the highest intensification in Raman. For instance, the bands at 1233 and 1471 cm^−1^ which are assigned to ring νCC stretching coupled to in plane deformations (δ) modes of the CH_3_ and COH fragments. The broad and medium bands at 453 and 554 cm^−1^ corresponds to a skeletal mode, while the other broad bands at 1306 and 1590 cm^−1^ are assigned to coupled vibrations of the νCC and δCOH modes^25^.

The main difference observed between glass and gold Raman spectra (Fig. 1c and 1d) is a group of narrow bands appearing in the 1200–1000 cm^−1^ range, as well as the bands at 852, 642 and 401 cm^−1^. The position and broadness of these bands suggest that they are due to the glucose residue of carminic acid. In order to confirm this, we have also registered the Raman of glucose on Au film (Fig. 1e). The good correspondence observed between the bands of carminic acid on gold with those of the Raman of glucose allowed us to suggest that the interaction of carminc acid with Au implies as well an approach of the Glu residue to the surface. On the other hand, these bands are attributed to νCO, νCC, δCOH, δCH and δCCC modes of the sugar moiety. However, the only sugar band that does not appear in the Raman of carminic is that at 930 cm^−1^, indicating that it is related to the anomeric C atom that links the anthraquinone moiety of carminic which is absent in the colorant. The carminic acid-gold interaction deduced from the spectra is also confirmed by the theoretical model resulting from the calculations (Fig. 1b), also revealing that an effective glucose residue metal surface interaction is taking place.

### Raman analysis of fibers

**Figure 2:**
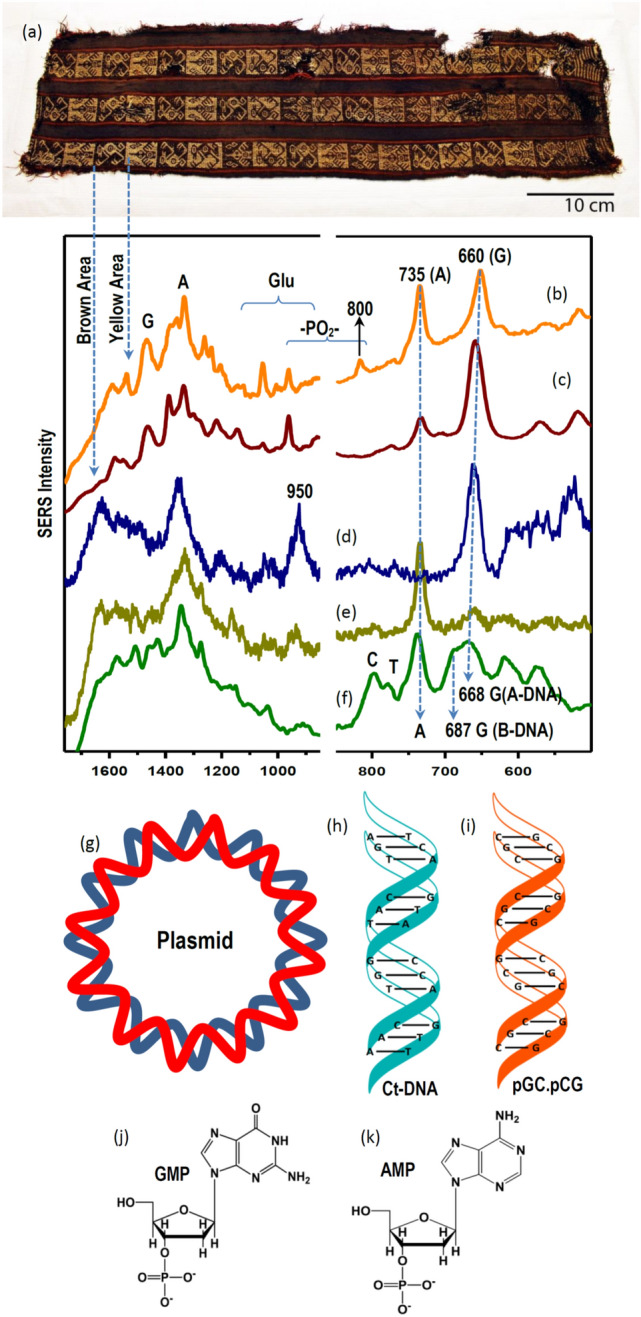
Image of the belt-bag from the Arica Culture (**a**). SERS spectra of colored fibers after adding Ag citrate nanoparticles to fibers extracted from the yellow (**b**) and brown (**c**) areas. SERS spectra of ct-DNA (**d**), pGC.pCG (**e**), and plasmid (**f**). Schemes of the plasmid (**g**), ct-DNA (**h**) and pGC.pCG (**i**), and structure of guanosin monophosphate (GMP) (**j**) and adenosine monophosphate (AMP) (**k**) by using Ag citrate nanoparticles.

**Figure 3:**
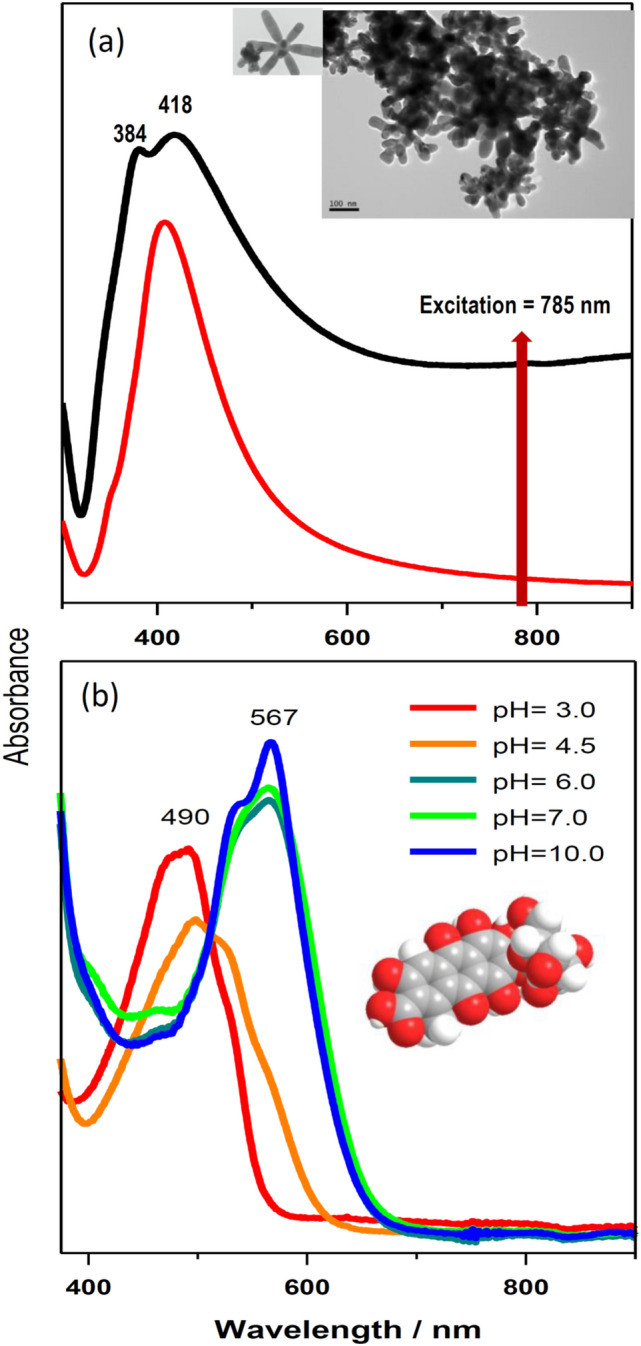
Extinction spectra of Ag nanostars (black) and AgNP obtained by reduction with citrate (red) (**a**). Inserted image: TEM images of Ag nanostars. UV–visible spectra of carminic acid and carminate at different pH (2 × 10^–5^ M) (**b**).

Figure 2 displays the SERS spectra registered in the analysis of the belt-bag shown in Fig. 2a. This belt-bag dated from the Formative Period corresponding to the Arica Culture and stored at the Museo Chileno de Arte Precolombino in Santiago de Chile. SERS spectra onto the fiber were measured by using AgNS nanoparticles. The advantage of these NPs is that they display a large extinction in the near infrared region (Fig. 3a, black line) due to the oblate arms existing in this NPs (TEM image inserted in Fig. 3a), thus rendering high SERS efficiency when using the 785 nm laser excitation. Conversely, spherical citrate nanoparticles exhibit a lower resonance in the near-infrared region (Fig. 3a, red line).

In particular, the SERS spectra were obtained from the yellow-orange and brown points (Fig. 2b and 2c). These spectra are dominated by some bands that cannot be found in the spectra registered on carminic acid reported in the literature^25^. This is the case of those bands appearing at 650–660 and 735 cm^−1^. There is strong evidence that these bands could actually be assigned to nucleobases, specifically ring breathing band of the nucleobases guanine (G) and adenine (A) due to the great similarity with the Raman spectra of these bases^34–36^. In addition, other bands of these nucleic bases can also be identified in the 1300–1500 cm^−1^ region, while other less intense ring breathing bands are observed at 770 and 800 cm^−1^ corresponding to the nucleobases thymine (T) and cytosine (C)^37, 38^. Similar spectra were also obtained from the analysis of many other textile materials from the Arica area in North Chile (results not shown). Moreover, bands appearing at similar wavenumber values were also identified in archaeological textiles dyed with cochineal that were original from Peru^7^.

The presence of nucleobase Raman bands in the SERS of these archaeological textiles led us to verify this by measuring the SERS of DNA materials. In this case citrate AgNPs and excitation at 514.5 nm were used. The use of these conditions leads to more intense spectra in these cases. Figure 2d, e and f display the Raman spectra of different nucleic acids adsorbed on Ag nanoparticles. The SERS spectrum of ct-DNA (Fig. 2e, see structure in Fig. 2h) is dominated by the A residue due to the higher affinity of this pyrimidine moiety for the metal^39, 40^, although a weak G signal is also observed at 660 cm^−1^. The bands corresponding to G are better observed in the SERS spectrum of the pGC.pCG polymer (Fig. 2d, see structure in Fig. 2i), where G predominates over C due to the higher affinity of the purine residues in comparison with the pyrimidine ones. Both A and G bands are simultaneously observed in the SERS of a plasmid, another DNA with a circular structure (Fig. 2f**,** see structure in Fig. 2g). In the latter spectrum a doublet is observed at 668 and 687 cm^−1^, corresponding to the ring breathing bands of G under A- and B-DNA coexisting structures in this polynucleotide^41, 42^. Thus, we have concluded that the presence of these nucleobases in historical textiles is attributed to the dyeing of fibers with raw cochineal extract, which was directly prepared from the insect containing biological residues from the insect body employed in the colorant fabrication.

Although bands of nucleic bases residue predominate on the spectra of textiles, other bands corresponding to other DNA component, such as phosphate group, can also be seen in the 800–1000 cm^−1^ spectral range. This is the case of the strong band at 950 cm^−1^ which is observed at 930 cm^−1^ in the SERS of pGC-pCG (Fig. 2d). Other bands in the 1500–1600 cm^−1^ range can be also assigned to the purine vibrations of the G residue.

Nucleic base bands are observed with a higher intensity in SERS spectra than protein or carbohydrate ones, also present in the cochineal extract. This is attributed to the higher affinity of purine and pyrimidine nucleobases for the metal^43^. The analysis of other complex biological materials by SERS reveals that nucleobases always display the strongest signals in non-labelled SERS analysis of biological material^44, 45^.

**Figure 4:**
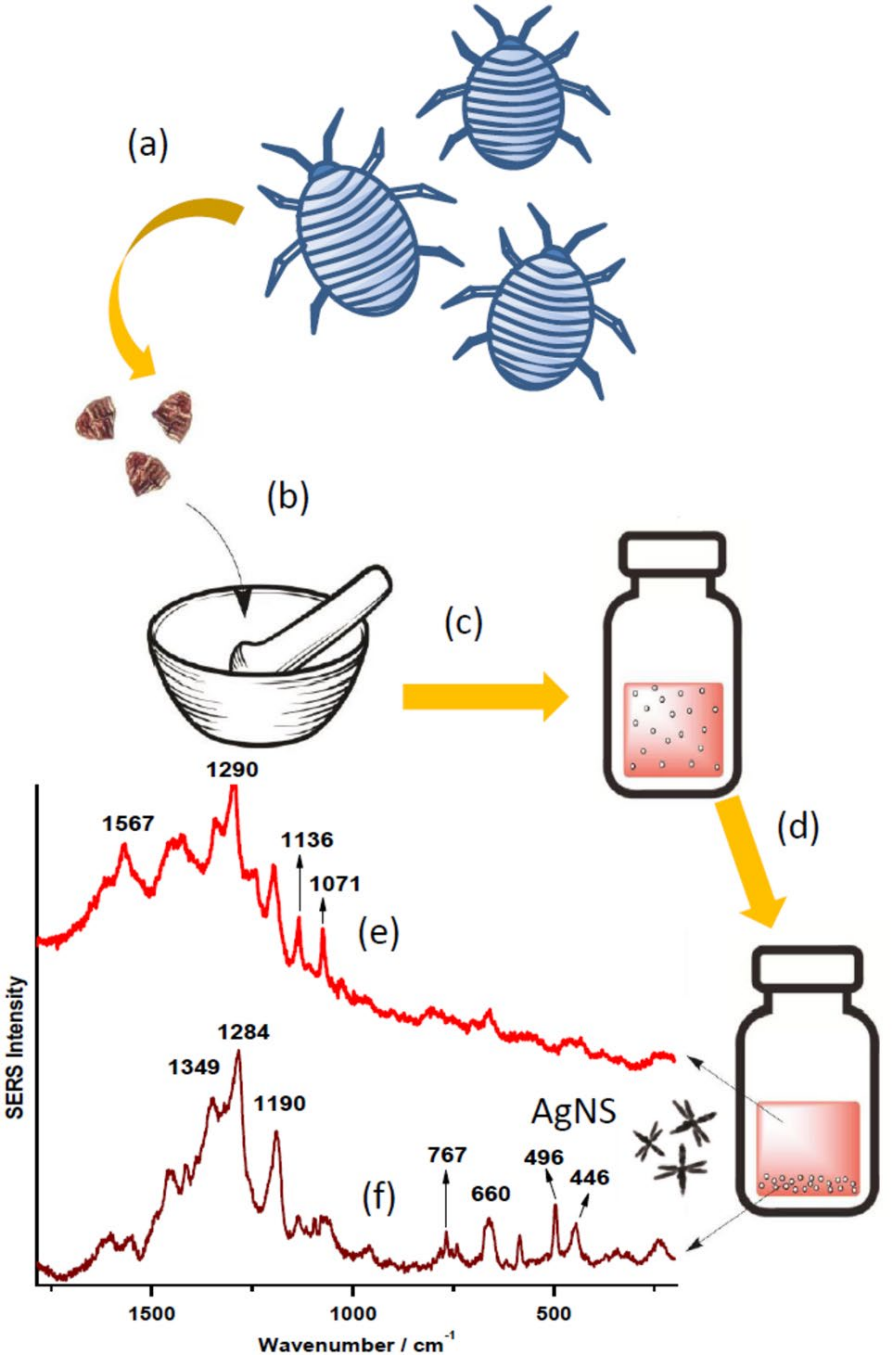
Extraction process followed to obtain the cochineal dye involving the following processes: drying (**a**), grinding (**b**), water suspension (**c**) and separation of supernatant and bottom residue (**d**). SERS spectrum obtained by using Ag nanostars and a sample obtained from the supernatant (**e**) and the bottom residue (**f**).

Replicas of the archaeological samples were prepared to further demonstrate that the presence of nucleic acids components in cochineal is due to the raw extract of cochineal insects. These samples were prepared by reproducing the methods employed in the past regarding the extraction of the cochineal colorant from the insect according to the scheme displayed in Fig. 4a-d. In order to better analyze the sample, the aqueous suspension obtained from the powdered insect extract was separated in two parts: the aqueous supernatant and the solid residue in the bottom. SERS spectra were measured from these two parts and are shown in Fig. 4e and f**,** respectively. SERS spectrum of the supernatant extract (Fig. 4e) show bands that can clearly be assigned to carminic acid at 1071, 1136, 1290 and 1567 cm^−1^, and that can also be seen in SERS spectra of the carminic acid previously reported^21, 25, 46^. However, the bottom residue gives rise to many bands that can be attributed to the biomolecules predominantly existing in this fraction. In particular, the band at 660 cm^−1^, due to G, and weaker bands at 737 and 767 cm^−1^, attributed to A and T^34, 47^, respectively. Moreover, the bands at 585 and 496 cm^−1^ could correspond to skeletal structural vibrations in nucleic acids^48^ or bending NH vibrations in nucleobases^49^, although some contribution from other related biological compounds such as proteins is also possible^50^. Furthermore, the broad band at 446 cm^−1^ is reported as a CO deformation in purine rings of nucleobases such as guanine or xanthin^39, 43^. The bands corresponding to phosphate moieties from nucleic acid chains^48^, seen in the 900–1100 cm^−1^ range also corroborate the presence of these types of biological species in cochineal raw material.

At higher wavenumbers, bands of the anthraquinone moiety of carminic acid are mixed to the purine and pyrimidine rings bands of nucleobases. Even though, the bands appearing at 1349, 1284 and 1190 cm^−1^, are due to carminic acid and correspond to those seen at 1344, 1294 and 1196 cm^−1^, in the aqueous supernatant fraction. Other bands at 1136 and 1071 cm^−1^ are due to the glucose residue in carminic acid also observed in the Raman analysis of the colorant (Fig. 1e). The shifts observed in some bands of the colorant are attributed to the presence of many other molecules that can affect its adsorption on the metal surface.

**Figure 5:**
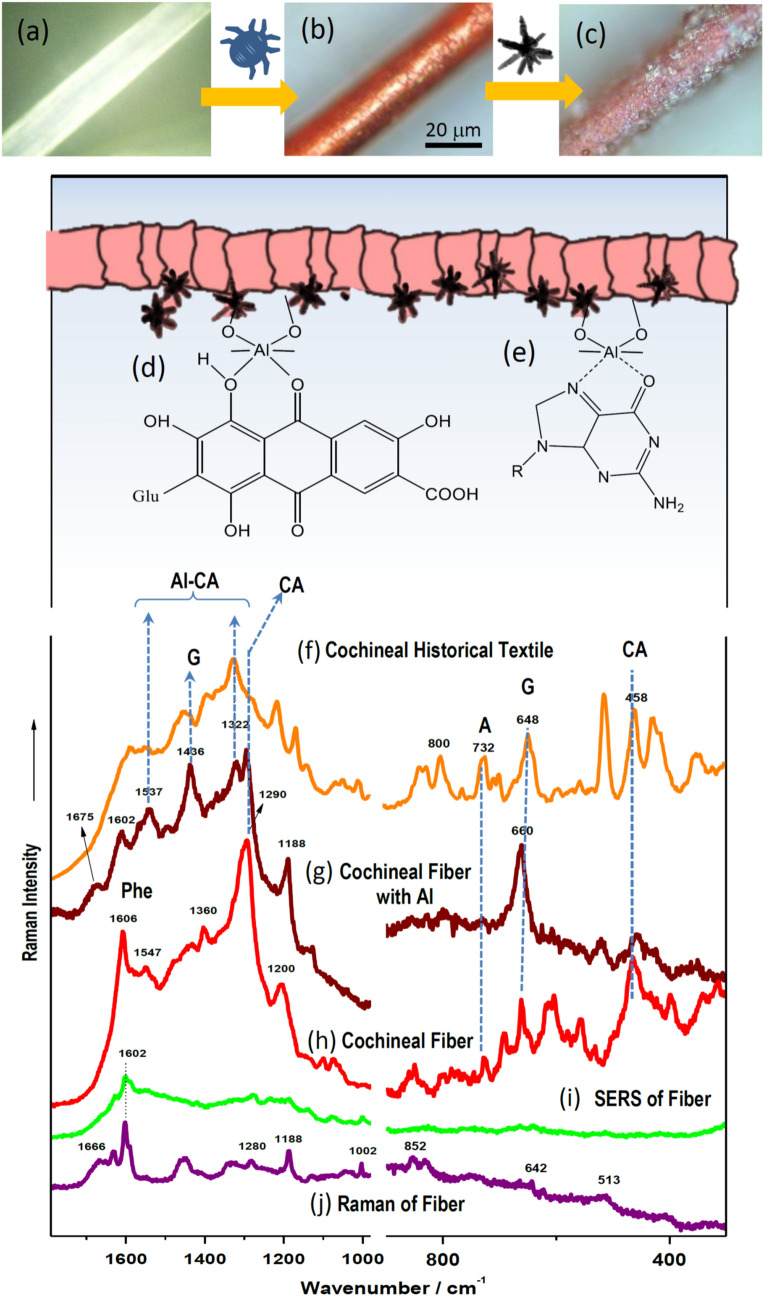
Steps followed to dye the commercial wool fiber (**a**) by the cochineal supernatant extract (**b**) and the Ag nanostars deposition to measure the SERS spectra (**c**). Schemes showing the Al-CA (**d**) and Al-G (**e**) interaction mechanisms deduced from the SERS spectra in mordanted cochineal dyes wool fibers. SERS spectrum obtained from the historical textile, in this case the orange fibers in the belt-bag of Fig. 2a (**f**); SERS spectrum of the cochineal dyed wool mordanted with Al_2_(SO_4_)_3_ (**g**); SERS spectrum of the cochineal dyed fiber without Al_2_(SO_4_)_3_ (**h**); SERS spectrum of non-dyed wool fiber (**i**) and Raman spectrum of the wool fiber (**j**).

The extracted cochineal colorant was then employed to dye commercial wool fibers in the fabrication of textile replicas. Figure 5 shows in the top panel the fibers before dyeing (Fig. 5a); cochineal dyed fiber (Fig. 5b) and the dyed fiber with AgNS deposited on the surface (Fig. 5c) employed to obtain the SERS spectra. Raman spectroscopy is one of the few techniques that allow the *in-situ* study of dyed fibers, but the extremely high fluorescence of cochineal makes necessary the use of SERS to quench this fluorescence. The SERS spectra were registered upon deposition of AgNS on wool fibers dyed with cochineal at two different conditions: directly on the fiber (Fig. 5h) and by using Al_2_(SO_4_)_3_ as mordant (Fig. 5g). The latter spectra were compared to the Raman and the SERS spectrum of non-dyed wool fibers (Fig. 5j and i). The Raman of the wool fiber displays typical bands corresponding mainly to the amino acid phenylalanine (1606, 1002, 852 cm^−1^); tryptophan (1547, 1360 cm^−1^); tyrosine (1615, 1188, 825 cm^−1^); cysteine (665, 513 cm^−1^), as well as bands corresponding to the polypeptide keratin backbone amide III and amide I at 1280 and 1666 cm^−1^, respectively.

SERS spectrum of directly dyed cochineal fibers (Fig. 5h) displays intense carminic bands at 1290 and 1200 cm^−1^, but also many of the ring breathing bands of nucleobases observed in the 800–600 cm^−1^ region, which are also observed in the SERS analysis of historical textiles (Fig. 5f, corresponding the latter to the orange areas of the belt-bag shown in Fig. 3a). In addition, intense structural bands from biomolecules are also seen below 600 cm^−1^.

The use of Al as mordant leads to a deep modification of the SERS of the resulting fiber, the presence of this metal induces a selective enhancement of the 660 cm^−1^ band due to the G residue (Fig. 5g), while other bands ascribed to skeletal vibrations below 500 cm^−1^ are also modified by the mordant effect of the metal. The specific interaction of G with Al could take place through the inner amide moiety in the purine ring (Fig. 5e). This interaction could also explain other changes observed in the high wavenumber region, such as the intensification of other bands attributed to the G residue at 1675, 1602 and 1537 cm^−1^, although shifted from their usual position in nucleic acids due to an effect of complexation with the metal in the mordant. Since the latter bands are mainly attributable to the C=O stretching of the pyrimidine ring coupled to ring vibrations and the stretching of C=N group in the imidazole ring^51^, we suggest that an interaction of Al with the ketonic oxygen and the N7 in the imidazole ring is taking place in the metallic complex (Fig. 5e**)**. This selective interaction of G with Al has been also recently described in the literature, involving a strong charge-transfer between Al to the C=O and the N7 atom of the G purine residue^52, 53^.

Al is then interacting very strongly with the carminic acid and thanks to this mordant interaction improves the fixation of the colorant to the wool fiber through the keto and phenol groups of the anthraquinone rings (Fig. 5d), as also deduced from the SERS analysis of this molecule onto metallic nanoparticles^25^. As a consequence of this strong interaction, a large shift of the 1290 cm^−1^ characteristic band of carminic to 1322 cm^−1^ is observed. The latter shift is very important in the identification of the presence of mordants in historical textiles, as similar effects can be observed in the analysis of many historical cochineal-dyed fibers. This is the case of the SERS of the historical textile fiber shown in Fig. 5f and others previously reported^7^. The 1290 cm^−1^ band is attributed to the C–OH deformations coupled to ring stretching vibrations in the anthraquinone ring I^25^ (Fig. 1). Therefore, the shift to higher wavenumbers is attributed to a strong interaction of carminic acid with Al.

However, the analysis of archaeological textiles still reveals a large variability regarding the bands intensity of G and A nucleobases, as well as other biomolecule characteristic bands depending on the color of the studied area. This is the case of the belt-bag analyzed in the present work, where different spectra were measured in the red, brown, yellow and orange areas (Fig. 2a) showing bands attributed to A, G, and carminic acid. In addition to all these elements of variability, it is necessary to mention that cochineal can have different colors and structures due to the possible ionization of the carboxylic acid and phenolic OH groups as reveal the UV–vis spectra registered for carminic at different pH (Fig. 3b). We suggest that different colors could be produced in the mordant process by changing the relative amount of metal, and other additives such are organic acids or ammonia, in relation to the raw cochineal material. As a result, different recipes could lead to different purple-red-orange hues observed in the same archaeological material^54^. Therefore, more investigation on the effect of the metal and the presence of biomolecules should be done in order to better interpret the resulting SERS spectra and correlate them to the analyzed archaeological samples.

## Conclusions

SERS spectroscopy has been successfully employed for the multiplexing identification of the different components integrating the cochineal colorant. This analysis was performed on the cochineal dyed archaeological textile samples and replicas using *in-situ* experiments that does not imply the colorant extraction. The regular Raman study of carminic acid indicates that the spectrum is dominated by the anthraquinone moiety signals, although Raman signals from the glucose residue have been obtained and identified when carminic acid is deposited on a gold thin layer. The interaction with metal surfaces leads to the relative intensity increasing of the glucose residue bands which is evidenced from the SERS spectrum of carminic acid and the theoretical representation of the dye-surface interaction. The SERS analysis of archaeological red dyed textiles demonstrated the existence of carminic acid, but also other biomolecules, mainly nucleic acids, in real archaeological textiles. The presence of nucleic acids in these samples was deduced from the appearance of nucleobase bands that can be mainly attributed to adenine and guanine, also detected in the raw cochineal colorant. This was further confirmed by obtaining cochineal dyed textile wool fibers according to ancient receipts. The effect of Al in mordant carminic acid leads to a significant structural change in the Raman spectrum of carminic acid involving the keto group as well as the adjacent C–OH groups. The complexation of Al also leads to specific interactions of the nucleobases with the wool in the fiber. In fact, guanine bands are further enhanced in SERS spectra because of the strong interaction established between Al and the purine residue through the C=O or pyrimidine ring and the N7 of imidazole ring. The results of the present work will be very useful in the identification of different molecules and metal complexes that may be forming part of the cochineal colorant found in archaeological materials.
